# Editorial: Tick-Host-Pathogen Interactions

**DOI:** 10.3389/fcimb.2018.00194

**Published:** 2018-06-15

**Authors:** Sarah I. Bonnet, Ard M. Nijhof, José de la Fuente

**Affiliations:** ^1^UMR BIPAR Institut National de la Recherche Agronomique-ANSES-ENVA, Maisons-Alfort, France; ^2^Institute for Parasitology and Tropical Veterinary Medicine, Freie Universität Berlin, Berlin, Germany; ^3^SaBio, Instituto de Investigación en Recursos Cinegéticos IREC-CSIC-UCLM-JCCM, Ciudad Real, Spain; ^4^Department of Veterinary Pathobiology, Center for Veterinary Health Sciences, Oklahoma State University, Stillwater, OK, United States

**Keywords:** ticks, host, pathogen, tick-borne pathogens, transmission, vector, tick-borne diseases

Ticks are important vectors of pathogens affecting human and animal health around the world. They act as vectors of several pathogens causing diseases of major concern including Lyme borreliosis, anaplasmosis, rickettsiosis, ehrlichiosis, tularemia, and tick-borne encephalitis (TBE) in humans, and babesiosis, theileriosis, and anaplasmosis in livestock. Therefore, a One Health approach is important for the effective prevention and control of tick-borne diseases (TBDs). Current tick control strategies rely essentially on the use of chemical acaricides, but their widespread use in livestock is associated with the selection of resistant tick populations and environmental contamination. In addition, socioeconomic and environmental changes affect tick distribution, with global incursions of TBDs. New approaches that are environmentally sustainable and control the threat posed by tick-borne pathogens (TBPs) are therefore needed. Tick-host-pathogen interactions drive vector competence for transmitted pathogens, which together with behavioral and environmental factors affect vectorial capacity (de la Fuente et al.). Therefore, the understanding of tick biology and the interactions between ticks, their hosts, and TBPs is essential for the identification of drivers for TBDs. In this research topic, we have gathered original research, review, perspective and opinion papers from scientists who have common interests in unraveling tick biology and tick-host-pathogen interactions for the development of new interventions for the control and prevention of TBDs.

A recent landmark in tick research was the publication of the first draft tick genome of *Ixodes scapularis*, the main vector of *Borrelia burgdorferi* in North America (Gulia-Nuss et al., 2016). Papers on how the availability of this genome and other advances made in genomics could be used within research on tick-host-pathogen interactions to eventually improve global health were included within this research topic (de la Fuente et al.; Grabowski and Hill). Examples of how genomic data can be deployed to identify and characterize metabolic pathways was presented in original research papers investigating changes in tick cell metabolism in response to *Anaplasma phagocytophilum* infection (Cabezas-Cruz et al.; Cabezas-Cruz et al.).

A comprehensive overview of tick-pathogen interactions and vector competence for bacteria, viruses, and protozoa was presented in a review by de la Fuente et al. It is remarkable that many vector-borne pathogens use similar strategies, such as remodeling of the cytoskeleton and manipulation of the vector immune response, to facilitate infection, multiplication, and transmission. A review on tick-pathogen interactions was included for *Babesia* species (Antunes et al.), and interactions between tick-borne viruses with both ticks and hosts were outlined in two other reviews (Kazimirova et al.; Papa et al.). In this research topic, Adams et al. presented the first dual reporter system for *B. burgdorferi*, a highly versatile tool for investigating pathogen transcription and gene regulation, both *in vitro* and in infected ticks. Lesser known *Borrelia* species associated with relapsing fevers in humans and mainly transmitted by soft ticks of the *Ornithodoros* genus were reviewed by Talagrand-Reboul et al. while emerging tick-borne viruses such as severe fever with thrombocytopenia syndrome virus (SFTSV), and factors leading to their emergence were reviewed by Mansfield et al.. Pruneau et al. used a comparative transcriptome profile to characterize the interactions between host and virulent and attenuated strains of *Ehrlichia ruminantium*, the causative agent of heartwater, to identify genes involved in pathogen virulence.

When taken up with an infected blood meal, pathogens first encounter the microbiome residing within the tick gut. Although still largely unexplored, Bonnet et al. reviewed the current knowledge on the role of non-pathogenic microbes in the gut and other tick tissues in pathogen transmission and tick biology with implications for the control of TBDs. The transcriptomics response to infection with the bacterial pathogen *Rickettsia rickettsii*, the causal agent of Rocky Mountain Spotted Fever, in the midgut of two *Amblyomma* tick species with different susceptibility to infection was described by Martins et al. and led to the selection of tick transcripts that might be implicated in the vector competence. Once pathogens cross the midgut barrier, they encounter components of tick innate immunity in the tick hemocoel. How different pathogens manage to evade the tick's innate immune system was the subject of a review by Sonenshine and Macaluso, and the role of Toll signaling in ticks in response to virus infections was addressed in a perspective article (Johnson). Components of the innate immunity in *Ixodes ricinus* ticks, the main vector of *B. burgdorferi* s.l. in Europe, were examined in more detail in two original research papers (Honig Mondekova et al.; Urbanova et al.). A review from Blisnick et al. also emphasized the importance of protease inhibitors in both tick biology and tick-borne pathogen transmission, being involved in tick feeding process but also in tick innate immune defense. These studies highlighted the mechanisms used by transmitted pathogens to evade tick innate immunity for infection while ticks respond to limit infection and preserve fitness.

Tick salivary glands and tick saliva play a pivotal role during tick feeding and pathogen transmission, and they were therefore a subject of several reviews within this research topic (Chmelar et al.; Mans et al.; Simo et al.). In addition, five original research papers investigated the sialotranscriptome and sialome of *Amblyomma sculptum* ticks (Esteves et al.), the sialotranscriptomics response of *Rhipicephalus bursa* ticks infected with *Babesia ovis* (Antunes et al.), the effect of salivary cystatin OmC2 from *Ornithodoros moubata* saliva on the host immune response (Zavasnik-Bergant et al.), and differences in the saliva protein profile of *I. scapularis* and *Amblyomma americanum* ticks stimulated to feed on different hosts (Tirloni et al.). The latter study showed that the salivary protein profiles differed between ticks of the same species that fed on different animals, an observation which for instance may have implications for studies looking at tick salivary antigens as anti-tick vaccine candidates. A research paper by Rodrigues et al. presented the relationship between *Amblyomma variegatum* salivary composition and regulation of host defenses. In another study, Zavasnik-Bergant et al. demonstrated the effect of salivary cystatin OmC2 from *O. moubata* on the host immune response through interaction with Cathepsins S and C.

Knowledge of tick genes differentially regulated in response to feeding or infection can be used to identify candidate tick protective antigens for the prevention and control of tick infestations and/or pathogen transmission through vaccination. The identification of tick protective antigens could be approached using different screening platforms (de la Fuente et al., 2016). A vaccinomics approach was applied to *Ixodes* ticks infected with *A. phagocytophilum* in an original research paper by Contreras et al. to identify and characterize candidate tick protective antigens. The current status and future prospects of vaccines targeting metazoan parasites, including ticks, was reviewed in a paper by Stutzer et al.

Upon transmission by ticks, TBPs need to evade the host immune response. An original research paper described the transcriptional profile of cutaneous immune responses to *I. ricinus*-transmitted tick-borne encephalitis virus (TBEV) during early stages of pathogen transmission (Thangamani et al.). The role of two *A. phagocytophilum* proteins, MSP4 and HSP70, in interactions with host cells was shown in another study (Contreras et al.), and analysis of the host cell response of human HL60 cells to *A. phagocytophilum* infection showed an increase in transcripts with a high proportion of alternatively spliced transcript events, thus providing important new fields of research (Dumler et al.). Host responses to tick infestations and tick resistance in cattle were the subjects of two complementary reviews (Robbertse et al.; Tabor et al.). A network analysis presented by Estrada-Peña and de la Fuente quantified the interactions between *I. ricinus* and transmitted *B. burgdorferi sensu lato* (s.l.) bacteria in the Western Palaearctic. The results showed that contrary to the prevailing paradigm, complex communities of vertebrates, which have large distribution ranges, instead of a few dominant vertebrates, support both *I. ricinus* and *B. burgdorferi* s.l. Associations of *I. ricinus* with its hosts and environmental niches that impact pathogen circulation was further evaluated in another study (Estrada-Peña et al.). It was demonstrated that the diversity of hosts increases the niche available for ticks and promotes the circulation of transmitted-pathogens. These results suggested that TBPs might manipulate ticks to occupy sub-optimal environmental niches (Estrada-Peña et al.). This manipulation can take place on an epigenetic level and there are increasing evidences that pathogens can induce transcriptional changes in both their vertebrate and tick hosts, thereby facilitating propagation of the pathogen. The potential implications of these tick-pathogen interactions for tick ecology were further discussed in an opinion paper (Cabezas-Cruz et al.).

Last but not least, recent advances in the study of tick-host-pathogen interactions have also led to the discovery of a wide variety of tick bioactive molecules that may be a source of novel therapeutics (Murfin and Fikrig).

As editors of the “Tick-host-pathogen interactions” Research Topic, we would like to acknowledge all contributing authors for providing insight into the exciting research that continues to improve our understanding of tick-host-pathogen interactions at molecular and ecological levels, and translation into new interventions for the control of TBDs.

## Author contributions

All authors listed have made a substantial, direct and intellectual contribution to the work, and approved it for publication.

### Conflict of interest statement

The authors declare that the research was conducted in the absence of any commercial or financial relationships that could be construed as a potential conflict of interest.
